# Supplementary material for: Evaluation of Maternal Genetic Background of Two Hungarian Autochthonous Sheep Breeds Coming from Different Geographical Directions
Source: Animals (Basel). 2022 Jan 18;12(3):218. doi: 10.3390/ani12030218 (PMC8833378; doi:10.3390/ani12030218)
Supplement: Supplementary file 1 [file animals-12-00218-s001.zip › Supplemantary Material 1 animals-1501954 Breed descriptions.pdf]

Breed descriptions (Tsigai and Cikta)

The Tsigai, also known by the names Cigája and Berke, is an old, independent long-tailed sheep breed originating in Asia Minor. The cloth factory and prosperous wool trade of Transylvania urged farmers to change to the Tsigai, which had a finer wool quality than the native coarse wool sheep bred here. The ancient Tsigai was not a primitive breed, but a highly developed multipurpose one [1]. During the 18th and 19th centuries, the golden age of the breed, it was unrivalled in the three primary uses of sheep: as a source of wool, milk and mutton. It never became dominant because its expansion was limited by the presence of the Hungarian Racka and the appearance of the Merino during the same period. At the end of the World War II, a small number of native animals that had been maintained made it possible to establish a gathered flock of 200 ewes in Karcag by 1950. This limited stock can be considered as a base and starting point of the national gene conservation program in the Tsigai breed. According to the current Hungarian breed-standard, the Tsigai is black or coffee-colored on the head and on the extremities (Figure S1). The fleece of lambs is sandy-grey, which turns after a few months to white. Some of the rams carry dark horns with 1.5 volutes. Ewes are polled or, rarely, carry rudimentary, sickle-like "goat-horns." The skin, gums, tongue and palate are slate-grey, as is the corneous matter. The skin is thin and elastic. The head is dry (lean or finely chiseled) and less convex from side view. The shoulders are firm; the withers are medium wide. The back and loin are straight, relatively long, and medium muscled. The loin is slightly slanting, medium long and wide, and can sometimes be short and sloped. The udder is well developed. The native Tsigai is extremely strong, hardy and resilient.

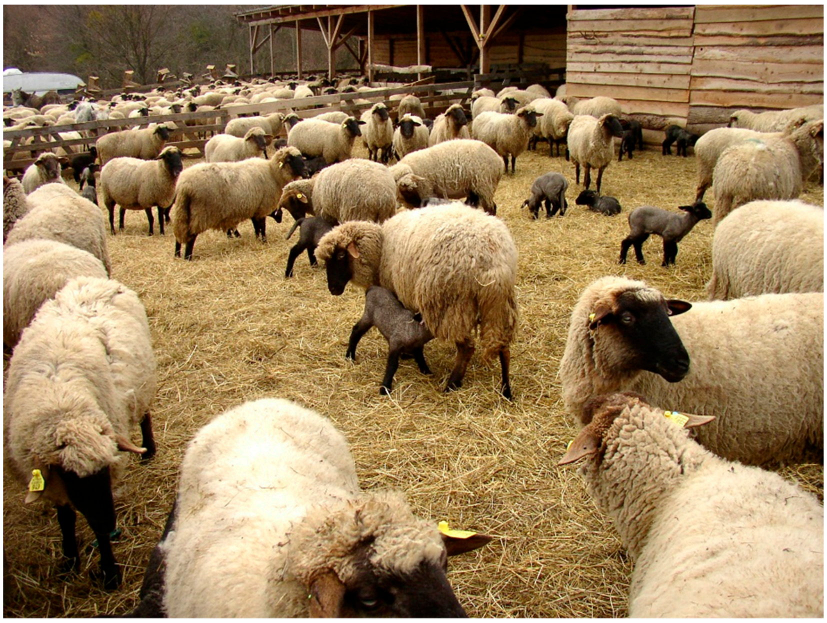

Figure S1. Tsigai ewes with their lams in winter corral, in Széltető (A. Gáspárdy, 2012)

The Cikta (known also under the name Hungarian Zaupel, Zibbe and Swabian Sheep of Counties Tolna and Baranya) reached southern Hungary with the Swabian settlers, around 1720. The animals had a much better wool quality than the local sheep, so that sheep keepers slowly

switched to this breed. Gradually, the breed became common throughout the whole Hungary [2]. The Cikta originated from the Zaupel sheep (Zaupelschaf). The Zaupel can be traced back to the Neolithic peat sheep (*Ovis aries palustris*; [3]), which, as a former companion of the Indo-Germanic tribes, populated areas stretching from the Balkans from 6500 BC.

By the middle of the 20th century the breed would have fallen into oblivion if the interest in autochthonous breeds of farm animals had not emerged. Due to a government regulation the remaining Cikta were collected within the country and relocated to the breeding institution of Bezzeg-puszta, in 1974. Since then, the state has supported the preservation of autochthonous sheep breeds in Hungary [4]. Cikta is characterized by non-seasonal breeding, and lambing twice a year was often provoked. They are very modest and able to withstand weather extremes. Like their close relatives, these races belonging to the threatened animal species. The Cikta is a breed of smaller body size, its height at the withers is about 58-60 cm, the ewes weigh 35-45 kg and the rams 45-55 kg (Figure S2). Head and limbs are covered with short, white hairs, claws and horns are waxy-yellow, the skin is unpigmented. Staple length is 20-24 cm, the fineness class is B/C and C/D. The shearing weight of ewes per year is 1.5-2 kg and of rams is 2.5-3.5 kg with a wool base of 65-70% [5].

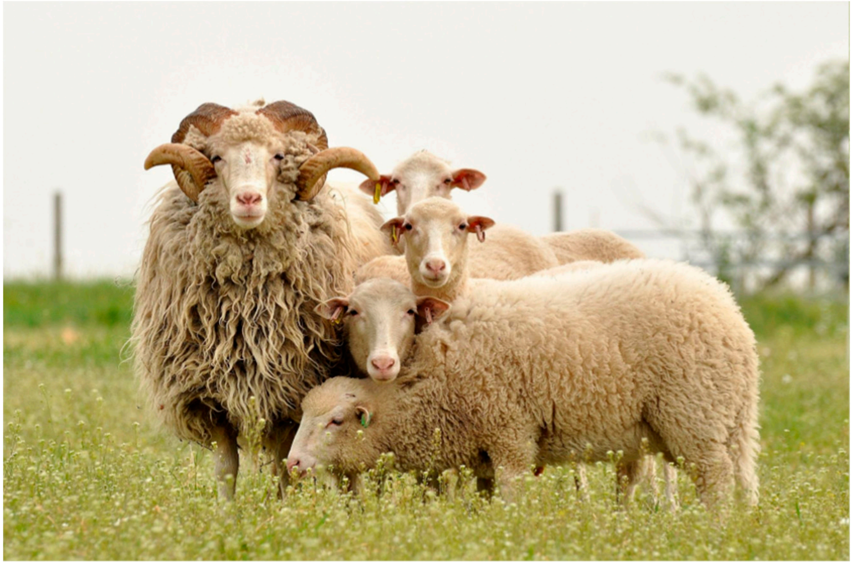

Figure S2. Cikta ram No. 11123605 with shorn mothers in Domoszló (Á. Balássy, 2013)

References

1. Gáspárdy, A. The Tsigai or Hungarian Berke. In *Living Heritage. Old Historical Hungarian Livestock*, 1st ed., Bodó, I., Ed.; Agroinform Publishing and Printing Ltd.: Budapest, Hungary, 2000; pp. 60–62.
2. Seibold, R. Ein historischer Zufall bewahrt das ungarische Zaupelschaf vor dem Aussterben: letzte Genreserve dieser Rasse im ungarischen Nagydorog (in German). *Unser Land* **1990**, *6(2)*, 43–44.
3. Mason, I.L. *Sheep breeds of the Mediterranean*. 1st ed., Food and Agriculture Organization of the United Nations. Commonwealth Agricultural Bureaux, Farnham Royal Bucks: Edinburgh, UK, 1967; pp.xix+215 pp.
4. Koppány, G. The Cikta sheep. In *Living heritage: Old Historical Hungarian Livestock*. 1st ed., Bodó, I., Ed.; Agroinform Publishing and Printing Ltd.: Budapest, Hungary, 2000; pp. 58–59.
5. Kovács, E.; Mitro, S.; Tempfli, K.; Zenke, P.; Maróti-Agóts, Á.; Sáfár, L.; Bali Papp, Á.; Gáspárdy, A. A specific selection programme is required in the autochthonous Cikta Sheep which is endangered by own frequent ARQ prion haplotype? *Landbauforsch Appl Agric Forestry Res* **2017**, *67(3-4)*, 141–146.
