# Supplementary material for: Evaluation of Maternal Genetic Background of Two Hungarian Autochthonous Sheep Breeds Coming from Different Geographical Directions
Source: Animals (Basel). 2022 Jan 18;12(3):218. doi: 10.3390/ani12030218 (PMC8833378; doi:10.3390/ani12030218)
Supplement: Supplementary file 1 [file animals-12-00218-s001.zip › Supplemantary Material 2 animals-1501954 Sampling places in Hungary.pdf]

Sampling places in Hungary

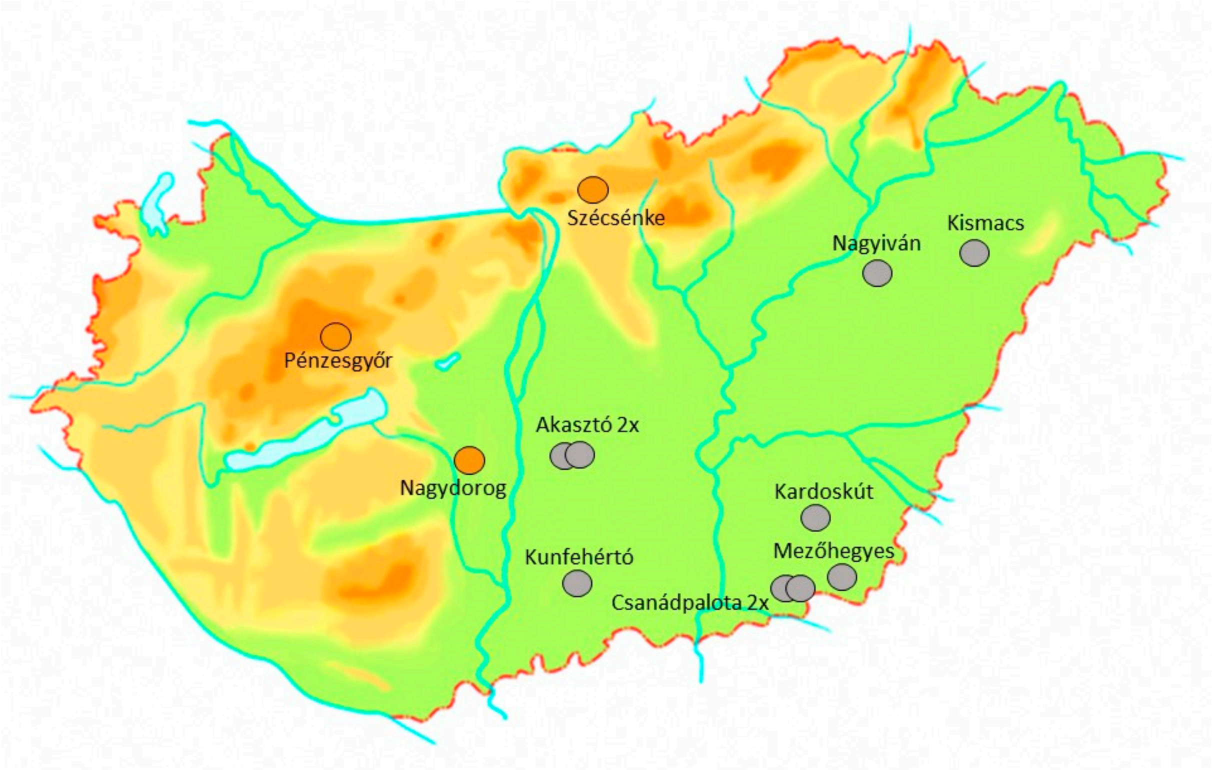

Figure S1. Presentation of sampling sites in Hungary. The location of the Tsigai stocks is indicated by gray circles and the location of the Cikta stocks by orange circles. The biological samples were obtained in 9 Tsigai flocks (Körös-Maros National Park - Kardoskút, Debrecen University - Kismacs, and seven private flocks in Akasztó 2x, Nagyiván, Csanádpalota 2x, Kunfehértó, Mezőhegyes) and in 3 Cikta flocks (Duna-Dráva National Park - Nagydorog, and two private farms in Pénzesgyőr and Szécsénke).
