# Supplementary material for: Evaluation of Maternal Genetic Background of Two Hungarian Autochthonous Sheep Breeds Coming from Different Geographical Directions
Source: Animals (Basel). 2022 Jan 18;12(3):218. doi: 10.3390/ani12030218 (PMC8833378; doi:10.3390/ani12030218)
Supplement: Supplementary file 1 [file animals-12-00218-s001.zip › Supplemantary Material 3 animals-1501954 Details of primers and PCRs.pdf]

Details of primers and PCRs

Table S1. Primers for CYTB and CR

Marker	Position	Primer sequences 5′-3′	Reference
CYTB	Forw-14159	TCATCATCATTCTCACATGGAATC	Meadows et al. [1]
	Rev-15298	CTCCTTCTCTGGTTTACAAGACCAG	
CR	Forw-15400	ACACCCAAAGCTGAAGTTCTAC	Gáspárdy et al. [2]
	Rev-16087	GTTGGTTTCACGCGGCATGGT	
	Forw-15983	AACTGCTTGACCGTACATAGTA	Hiendleder et al. [3]
	Rev-592	AGAAGGGTATAAAGCACCGCC	

A primer pair designed for the cytochrome b coding region (14,159–15,298) after Meadows et al. [1] consisted of CYTB-F 5′-GTCATCATCATTCTCACATGGAATC-3 ′and CYTB-R 5′-CTCCTTCTCTGGTTTA CAAGACCAG-3′ and applied following the amplification protocol presented here: an initial cycle of 94°C for 30 s, 6 cycles of 30 s at 94°C, 30 s at 54°C, 45 s at 72°C, 6 cycles of 30 s at 94°C, 30 s at 53°C, 45 s at 72°C, 21 cycles of 30 s at 94°C, 30 s at 52°C, 45 s at 72°C; and a final extension step 72°C for 7 min.

Two primer pairs were applied to amplify the sequence of control region: a first pair for the beginning of the region as described by Gáspárdy et al. [2] and a second pair as described by Hiendleder et al. [3].

First, the following amplification protocol was used for the newly designed primers (MtOA_F15400 5′-ACACCCAAAGCTGAAGTTCTAC-3′ and MtOA_R16087 5′-GTTGGTTTCACGCGGCATGGT-3′): an initial cycle of 94°C for 20 s, followed by 34 cycles of 94°C for 30 s, 62°C for 30 s, 72°C 45 s, and a final extension step 72°C for 7 min. The expected PCR product size was 688 bp. The second amplification protocol was used as follows (MtOA_F15983 5′-AACTGCTTGACCGTACATAGTA-3′ and MtOA_R592 5′-AGAAGGGTATAAAGCACCGCC-3′): an initial cycle of 94°C for 30 s, 6 cycles of 30 s at 94°C, 30 s at 54°C, 45 s at 72°C, 6 cycles of 30 s at 94°C, 30 s at 53°C, 45 s at 72°C, 21 cycles of 30 s at 94°C, 30 s at 52°C, 45 s at 72°C; and a final extension step 72°C for 7 min. Aligned and trimmed CR sequences corresponded to positions 15437–16616 on the reference sequence (AF010406 [3]).

Table S2. Composition of the PCR mixture.

Reagent	Amount
DreamTaq™ Green PCR Master Mix	5 μl
Primer mix (forward and reverse)	1-1 μl (10 μM)
DNA	1-10 μl (approx. 10 ng/μl)
BSA (Bovine Serum Albumin)	1μl (20 mg/ml)
PCR grade water	to volume
Total volume	25 μl

References

1. Meadows, J.R.S.; Li, K.; Kantanen, J.; Tapio, M.; Sipos, W.; Pardeshi, V.; Gupta, V.; Calvo, J.H.; Whan, V.; Norris, B.; Kijas, J.W. Mitochondrial sequence reveals high levels of gene flow between sheep breeds from Asia and Europe. *J Hered* **2005**, *96*, 494–501. DOI: 10.1093/jhered/esi100
2. Gáspárdy, A.; Berger, B.; Zabavnik-Piano, J.; Kovács, E.; Annus, K.; Zenke, P.; Sáfár, L.; Maróti-Agóts, Á. Comparison of mtDNA control region among descendant breeds of the extinct Zaupel sheep revealed haplogroup C and D in Central Europe. *Vet Med Sci* **2021**, First published: 22 July 2021. DOI: 10.1002/vms3.585
3. Hiendleder, S.; Lewalski, H.; Wassmuth, R.; Janke, A. The Complete Mitochondrial DNA Sequence of the Domestic Sheep (Ovis aries) and Comparison with the Other Major Ovine Haplotype. *J Mol Evol* **1998**, *47*, 441–448. DOI: 10.1007/PL00006401
