# Supplementary material for: Evaluation of Maternal Genetic Background of Two Hungarian Autochthonous Sheep Breeds Coming from Different Geographical Directions
Source: Animals (Basel). 2022 Jan 18;12(3):218. doi: 10.3390/ani12030218 (PMC8833378; doi:10.3390/ani12030218)
Supplement: Supplementary file 1 [file animals-12-00218-s001.zip › Supplemantary Material 4 animals-1501954 Number of polymorphic sites.pdf]

Number of polymorphic sites

Table S1. Sample size and number of polymorphic positions for CYTB and CR

Parameter	Tsigai	Cikta	total
Number of individuals/sequences			
CYTB	134	68	202
CR	130	69	199
Number of polymorphic sites/mutations			
CYTB	35	21	56
CR	162	108	270
Average number of mutations per individual			
CYTB	0.261	0.309^{NS}	0.277^{NS}
CR	1.246	1.565^{NS}	1.357

It can be seen that the number of mutations observed shows a positive relationship with the number of individuals included in the study. But the average number of mutations per individuals is inversely proportional to the sample size according to the breeds. Their values did not result in significant difference between breeds neither for CYTB (one-sided difference test between proportions, p = 0.476, NS) nor for CR (p = 0.427, NS). The 5-fold difference in the individual mutation rates of the two sequences in favor of CR either was not statistically significant (p = 0.160, NS).
